# Changes in public health preparedness services provided to local health departments by regional offices in North Carolina: a comparison of two cross-sectional studies

**DOI:** 10.1186/1756-0500-7-319

**Published:** 2014-05-28

**Authors:** Catherine V Donovan, Milissa Markiewicz, Jennifer A Horney

**Affiliations:** 1 Department of Epidemiology, University of North Carolina Gillings School of Global Public Health, Chapel Hill, NC 27599, USA; 2 North Carolina Institute for Public Health, University of North Carolina Gillings School of Global Public Health, Chapel Hill, NC 27599, USA

**Keywords:** Public health, Preparedness, Regionalization

## Abstract

**Background:**

In 2011, seven decentralized Public Health Regional Surveillance Teams (PHRSTs) were restructured into four centralized Public Health Preparedness and Response (PHP&R) regional offices to realign preparedness priorities and essential services with appropriate infrastructure; field-based staff was reduced, saving approximately $1 million. The objective of this study was to understand the impact that restructuring had on services provided to local health departments (LHDs) throughout North Carolina.

**Methods:**

A survey to document services that regional offices provide to LHDs in North Carolina was administered by the North Carolina Preparedness and Emergency Response Research Center in 2013. The results were compared to a similar survey from 2009, which identified services provided by regional teams prior to restructuring.

**Results:**

Of 69 types of assistance, 14 (20%) were received by 50% or more LHDs in 2012. Compared to 2009, there was a significant decrease in the proportion of LHDs receiving 67% (n = 47) of services. The size of the region served by regional offices was shown to inversely impact the proportion of LHDs receiving services for 25% of services. There was a slight significant decline in perceived quality of the services provided by regional teams in 2012 as comparison to 2009.

**Conclusions:**

Following a system-wide review of preparedness in North Carolina, the state’s regional teams were reorganized to refine their focus to planning, exercises, and training. Some services, most notably under the functions of epidemiology and surveillance and public health event response, are now provided by other state offices. However, the study results indicate that several services that are still under the domain of the regional offices were received by fewer LHDs in 2012 than 2009. This decrease may be due to the larger number of counties now served by the four regional offices.

## Background

Since 2002, federal funding for public health preparedness has been provided by the Centers for Disease Control and Prevention (CDC) to state, local, tribal, and territorial public health departments through the Public Health Emergency Preparedness (PHEP) cooperative agreement. PHEP cooperative agreements are the main source of funding that state health departments use to develop and maintain their ability to effectively respond to public health threats, including infectious diseases, natural disasters, and biological, chemical, nuclear, and radiological events [1]. These funds, a total of more than $9 billion so far, have also been supplemented by emergency response grants to address specific events, such as the 2009 H1N1 novel influenza A pandemic.

Many states have used PHEP funds, in part, to create and support preparedness regions with the goal of coordinating local efforts, consolidating services, and supplementing local government agencies [2]. By 2006, most states had established preparedness regions, with over half of these regions created after 2001[3]. North Carolina was among those states that created preparedness regions following the terrorist attacks of September 11, 2001. In December 2001, seven Public Health Regional Surveillance Teams (PHRSTs) were established to provide a range of services to a designated regional grouping of local health departments (LHDs), ranging in number from 7 to 17, with an average of 12. PHRSTs were originally designed to include a medical officer, an industrial hygienist, a nurse consultant, and an administrative specialist. Over time, the composition of some teams evolved to include a physician and an environmental health specialist. The seven teams also shared three pharmacists and had access to a veterinary medical response specialist as well as laboratory services.

In July 2009, the North Carolina Preparedness and Emergency Response Research Center (NCPERRC) surveyed preparedness coordinators (PCs) at LHDs to better understand how the regional teams contributed to local emergency preparedness and response capacity. The study found that a core package of 26 services was being provided to over 75% of LHDs in six broad categories of assistance: communication and liaison, exercises, epidemiology and surveillance, planning, consultation and technical assistance, and H1N1 outbreak response [4].

A strategic planning process was conducted by the North Carolina Division of Public Health (NCDPH) to realign preparedness priorities and essential services with appropriate infrastructure. The seven PHRSTs were consolidated into four Public Health Preparedness and Response (PHP&R) regional offices in 2011. Some of the services that had previously been provided by the PHRSTs were redistributed within NCDPH, including epidemiology and surveillance services, which were moved to the Communicable Disease Branch (CDB) and the Occupational and Environmental Epidemiology Branch (OEEB). The reorganized regional offices were shifted from local governance (i.e., decentralized) to regional offices of NCDPH’s PHP&R Branch (centralized), and budget and staff were reduced by approximately one-third. Currently, four regional offices, each comprised of a planning consultant, trainer/exercise facilitator, industrial hygienist, and pharmacist, provide services to a designated regional grouping of LHDs ranging from 11 to 31, with an average of 22.

The potential implications of the centralization of public health services on both operations and outcomes have been explored extensively by the public health systems and services research literature [5]. More centralized public health systems may be more effective and efficient at providing services due to the benefits of coordinating activities across jurisdictions [6]. For example, in a study of public health organizations in the largest metropolitan areas of the U.S., centralized jurisdictions provided 13% more public health services that decentralized or mixed jurisdictions [7]. However, more decentralized systems may be more entrepreneurial, and may also have a denser local network of non-public health organizations (e.g., schools, faith based organizations, senior centers, or youth organizations) working towards health improvement, particularly of certain groups like adolescents or seniors [8].

To better understand how the centralized regional offices now support LHDs in their preparedness efforts, the 2009 NCPERRC survey was re-administered to PCs at LHDs in February 2013. In both the 2009 and 2013 surveys, PCs were selected to complete the survey because of their close collaboration with the regional offices.

## Methods

The original 2009 NCPERRC survey included 80 services that were drawn from the PHRST operations manual and annual contract agreement addenda between NCDPH and LHDs [4]. These services were divided into seven categories of assistance: communication and liaison, exercises, epidemiology and surveillance, planning, consultation and technical assistance, training, and public health event response. An eighth category on H1N1 outbreak assistance was also included. The 2009 survey was reviewed by two PHRST staff and two PCs, and then piloted by a group of four former PCs, two PHRST staff, and two NCDPH employees. The final electronic survey was administered to PCs at LHDs in North Carolina in August 2009, and asked about services received in the previous year.

A near-identical survey (minus the set of H1N1 outbreak assistance services that were no longer relevant, reducing the total to 69 services), was re-administered to PCs in February 2013. For each of the seven categories, respondents answered questions about the specific types of services their LHD received from their regional office during the period from July 1 – December 31, 2012. While the survey was administered in 2013, it asked questions regarding services provided in 2012, and will hereafter be referred to as the 2012 survey.

In addition to the seven categories, respondents were asked to rate the overall quality of services their LHD received from their regional office. Quality was measured on a Likert- type scale from 1 (*poor*) to 4 (*exceptional*). Respondents were also asked to select from a drop-down menu the three most important (in their opinion) categories of service their LHD received from their regional office and the three most frequently requested categories of service. Finally, respondents were asked to provide any open-ended comments on regional office services they wished to share.

The 2012 survey was created using Qualtrics v2013 (Qualtrics, Provo, UT), an online survey software, and a link to the survey was sent via e-mail to all PCs in the state’s 85 LHDs and the Health and Medical Division of the Eastern Band of Cherokee Indians (EBCI). In LHDs where the PC position was vacant or had a very recent hire, health directors, nursing supervisors, or health educators at the LHD received the link and completed the survey. Three reminder e-mails were sent and one phone call was made to non-responders. This research was approved by the institutional review board of the University of North Carolina at Chapel Hill (09–0523).

To quantify the types of assistance received by each LHD, survey data were exported from Qualtrics into SAS v9.2 (SAS Inc, Cary, North Carolina). SAS was used to calculate the percentage of LHDs that reported receiving assistance in each of the seven categories. Fisher’s exact tests with two-sided p-values were used to compare the proportion of respondents receiving each type of assistance in 2012 to the proportion that had received the same type of assistance in 2009. Results were stratified by region, and a Cochran-Armitage trend test with one-sided exact p-values was used to determine if the number of LHDs receiving assistance in each category decreased according to an increase in the number of LHDs covered by a regional office. Mantel-Haenszel chi square tests were used to compare the mean quality score from 2012 to 2009, and to test for a shift in mean quality score between regions. Pearson’s chi square tests were used to test for an association between year of the survey and both the most important services provided and the most frequently requested. Associations and differences in proportions and trends were considered significant if reported p-values were less than 0.05.

In both the 2009 and 2013 surveys, LHD respondents were also asked to provide information about trainings received from regional offices during the previous six months. Trainings were categorized into ten categories: respiratory tract infection, exercises, communication, Strategic National Stockpile (SNS)/CHEMPACK, general emergency preparedness and response, public health preparedness capabilities, radiological hazards, suspicious substances, vulnerable and at risk populations, and other.

## Results

Eighty-five LHDs completed the survey, plus the Health and Medical Division of the EBCI, for a 100% response rate. Of the 86 respondents, 80% included the title of “preparedness coordinator” in their job title.

### Services provided in 2012

In 2009, a core package of 26 services was received by 75% or more of LHDs. In the current study, only six services were received by 75% or more of LHDs. Due to the decreased number of services provided in 2012, we reduced our reporting threshold to 50%. Table 1 lists the services that 50% or more of the LHDs reported receiving in the period from July 1, 2012 – December 31, 2012. Of the 69 potential types of assistance listed in the survey, 14 (20%) were received by at least half of the LHDs. The communication and liaison category had the highest number of services received by at least 50% of LHDs (8/12, 67%). In the public health event response category there were no services that were delivered to more than half the LHDs.

**Table 1:** Services received by 50% or more of local health departments in 6 categories of assistance

**Type of service**	**% of LHDs receiving service**
Planning assistance (n = 6)	
Strategic National Stockpile plan development/refinement	95
Communication and liaison assistance (n = 12)	
Hold regular meetings of preparedness Coordinators in the region regarding public health preparedness and response activities	99
Test a telecommunications system	94
Facilitate relationships with local/regional preparedness partners	76
Serve as a liaison between local/regional public health and NC DPH	73
Represent local/regional public health preparedness at meetings held by partner agencies	72
Communicate relevant information from state and national preparedness meetings or professional conferences to the LHD	70
Share contact lists of regional response partners	66
Assist with access to and use of web emergency operations center	65
Training assistance (n = 2)	
Provide information on training opportunities available online or from other agencies	90
Epidemiology and surveillance assistance (n = 10)	
Promote the use of NC Health Alert Network by LHDs	59
Facilitate access to NC Health Alert Network and/or Epidemic Information Exchange	56
Exercise assistance (n = 15)	
Provide guidance to the LHD about exercises	81
Consultation and technical assistance (n = 10)	
Industrial hygiene	51

*Abbreviations*: *NC DPH* North Carolina Division of Public Health, *LHD* Local Health Department.

### Changes in services

Of the 69 services, 61 (88%) were provided to fewer LHDs in 2012 than in 2009. Three services were provided to more LHDs in 2012: assistance with the development of a training plan for LHD staff (change from 36 to 37%), providing information on training opportunities available online or from other agencies (change from 87 to 90%), and meth lab decontamination (change from 2 to 5%). None of these improvements were statistically significant. Forty-six services (67%) showed a significant decrease from 2009.

Among the types of planning assistance received by LHDs, three of the six services decreased significantly between 2009 and 2012. Four types of communication and liaison assistance decreased significantly. Half or more of the services in the categories of consultation and technical assistance and public health event response decreased significantly in 2012, as did all of the services in epidemiology and surveillance. Table 2 lists all services with significant decreases. Services which remain the primary responsibility of the regional offices after the consolidation are indicated to clearly separate them from services which are now primarily the responsible of other state offices, including NCDPH’s CDB and OEEB.

**Table 2:** Services that were received by significantly fewer local health departments in 2012, compared to 2009

**Type of service**	**% LHDs receiving service in 2009**	**% LHDs receiving service in 2012**	p-value*
Planning assistance			
Assistance with pandemic influenza planning**	59	13	<0.001
Assistance with isolation and quarantine planning**	87	27	<0.001
Assistance with risk communication planning**	63	38	0.002
Communication and liaison assistance			
Facilitating relationships with local/regional preparedness partners**	93	76	0.003
Communicating information from public health epidemiologists in the region**	79	41	<0.001
Communicating relevant information from state and national preparedness meetings or professional conferences**	92	70	<0.001
Assistance with community preparedness education**	74	43	<0.001
Epidemiology and surveillance assistance (n = 10)			
Share NC DETECT data	63	30	<0.001
Promote the use of NC DETECT	65	46	0.017
Share NC HAN alerts	80	45	<0.001
Promote the use of NC HAN	89	59	<0.001
Facilitate access to NC HAN and/or Epi-X?	82	56	<0.001
Assist with an epidemiological investigation	54	21	<0.001
Review or edit epidemiology data collection instruments	43	16	<0.001
Analyze or assist with the analysis of epidemiological data	36	13	<0.001
Facilitate GIS data collection and/or use of GIS equipment	32	8	<0.001
Support/promote epi team	47	20	<0.001
Exercise assistance (n = 15)			
Provide guidance to the LHD about exercises**	99	81	<0.001
Plan a single-county/district exercise for the LHD**	44	23	0.005
Facilitate an after action review meeting for the LHD**	48	18	<0.001
Assist with the development of an after action report for the LHD**	62	28	<0.001
Assist with the development of an IP or CAP for the LHD**	60	33	<0.001
Assist with the implementation of an IP or CAP for the LHD**	77	24	<0.001
Plan a multi-county exercise for the region**	95	9	<0.001
Provide a multi-county exercise scenario for the region**	91	14	<0.001
Moderate or facilitate a multi-county exercise for the region**	89	13	<0.001
Assist with the development of an after action report for the region**	84	19	<0.001
Assist with the development of an IP or CAP for the region**	88	18	<0.001
Assist with the implementation of an IP or CAP for the region**	77	14	<0.001
Consultation and technical assistance (n = 9)			
Industrial hygiene**	84	51	<0.001
Use of personal protective equipment**	76	39	<0.001
Infection control	68	22	<0.001
Interpretation of state or national guidance	73	17	<0.001
Pharmacy-related issues	58	34	0.002
Public health event response (n = 15)			
Interpretation of local, state, or national guidance related to the event(s)	57	13	<0.001
Investigation of incidents or events in cooperation with response partners	54	10	<0.001
Development and/or refinement of epidemiological investigation tools	54	7	<0.001
Implementation of event control measures in cooperation with response partners	35	0	<0.001
Incident/event long-term follow-up	30	3	0.009
Deployment of SNS	16	0	0.031
Development and/or distribution of communication materials	41	0	<0.001

*Abbreviations*: *LHD* Local Health Department, *NC DETECT* North Carolina Disease Event Tracking and Epidemiologic Collection Tool, *NC HAN* North Carolina Health Alert Network, *Epi-X* Epidemic Information Exchange, *GIS* Geographic Information System, *IP* Improvement Plan, *CAP* Corrective Action Plan, *SNS* Strategic National Stockpile.

^*^Fisher’s two-sided exact p-value.

**Services that remain the responsibility of the reorganized regional offices. Note that other services are now primarily provided by other programs in the North Carolina Division of Health.

### Variation in services by region

The proportion of LHDs receiving a specific type of assistance varied by region, and in 25% (n = 17) of cases was significantly associated with the size of the region. Among 25% of the 69 services, the number of LHDs covered by a regional team was significantly inversely associated with the proportion of LHDs in the region who received that type of assistance. Table 3 lists the 17 services whose proportion decreased with increasing region size. This association with region size was shown in four of the seven categories: planning, communication and liaison, training, and exercises, with the largest number of services that displayed this trend occurring in the exercises category (n = 12).

**Table 3:** Services that decreased significantly with an increase in region size in 2012

	Percent of LHDs receiving each type of service by region*				**Cochran Armitage trend test**	
**Type of service**	**CRI**	**Western**	**Eastern**	**Central**	**Z score**	**p-value**^**Ɨ**^
Planning assistance (n = 6)						
Isolation and quarantine	36	31	48	7	-1.97	0.029
Risk communication	73	38	29	34	-1.97	0.028
Communication and liaison assistance (n = 12)						
Facilitate relationships with local/regional preparedness partners	91	94	78	57	-2.90	0.001
Represent local/regional public health preparedness at meetings	82	81	77	57	-1.88	0.032
Training assistance (n = 2)						
Assist with the development of a training plan for LHD staff	55	50	39	23	-2.15	0.019
Exercise assistance (n = 15)						
Plan a single-county/district exercise for your LHD	45	47	26	0	-3.85	<0.001
Provide a single-county/district exercise scenario for your LHD	45	41	44	3	-3.05	0.002
Moderate or facilitate a single-county/district exercise for your LHD	40	41	35	7	-2.64	0.005
Evaluate a single-county/district exercise for your LHD	30	47	35	10	-2.05	0.024
Facilitate an after action review meeting for the LHD	50	41	9	3	-4.03	<0.001
Assist with the development of an AAR for the LHD	70	53	17	10	-4.28	<0.001
Assist with the development of an IP or CAP for the LHD	70	53	30	13	-3.77	<0.001
Assist with the implementation of an improvement plan IP or CAP for the LHD	50	53	22	3	-3.94	<0.001
Provide a multi-county exercise scenario for the region	33	12	22	0	-2.46	0.012
Assist with the development of an AAR for the region	67	12	22	7	-3.26	<0.001
Assist with the development of an IP or CAP for the region	56	12	22	7	-2.70	0.005
Assist with the implementation of an IP or CAP for the region	44	6	17	7	-2.08	0.024

*Abbreviations*: *LHD* Local Health Department, *AAR* After Action Report, *IP* Improvement Plan, *CAP* Corrective Action Plan.

*Number of LHDs covered by each region: CRI = 11, Western = 19, Eastern = 25, Central = 31.

^Ɨ^Cochran-Armitage one-sided exact p-value.

### Trainings provided

The LHD respondents listed 271 trainings provided by the regional offices. The trainings were categorized as follows: SNS/CHEMPACK (20%, n = 54), other (18%, n = 49), communications (14%, n = 38), exercises (13%, n = 36), general emergency preparedness and response (13%, n = 35), public health preparedness capabilities (9%, n = 24), radiological hazard (4%, n = 12), respiratory infection (4%, n = 10), suspicious substance (3%, n = 7), and vulnerable/at risk populations (2%, n = 6). Of these, general emergency preparedness, SNS/CHEMPACK, communications, and respiratory infection each represented a decreased proportion of the total number of trainings compared to 2009. Exercises and other trainings (including “progress check” training, a training required for LHD reporting to the North Carolina Division of Public Health on the use of PHEP funds) each represented an increased proportion of the total number of trainings, compared to 2009. Public health preparedness capabilities, radiological hazard, suspicious substance, and vulnerable/at risk populations were new training categories in 2012. Because LHDs in the same region would likely have attended the same training, these 271 trainings are not unique.

### Quality of services

Overall, LHD respondents rated the quality of assistance received from the regional offices as good (median = 3.0/4.0). This was similar to feedback from the 2009 survey, where the median quality rating was also 3.0. However, the mean quality score in 2012 (2.9) was significantly lower than in 2009 (3.3), indicating that the quality of assistance received from regional offices (as perceived by respondents) had declined (p < 0.0001). In 2009, the majority of respondents (48%) rated quality of PHRST assistance as exceptional (4.0/4.0). In 2012, the majority (39%) rated the quality of regional office assistance as good (3.0/4.0). There was no significant trend by region, indicating that quality of assistance was not associated with region size (p = 0.69).

### Importance and frequency of services

The three most important and most frequently requested types of assistance received by LHDs from regional offices, as reported by respondents, were planning, training, and consultation and technical support. There was a significant association (p = 0.03) between survey year and most important type of assistance. In comparison to 2009, planning increased in importance in 2012 (change from 26 to 40% of respondents who listed it as most important); training also increased in importance (change from 13 to 22%), while consultation and technical support decreased in importance (change from 32 to 18%). In 2009, exercises were ranked as one of the top three most important services; this category decreased in 2012 from 17 to 8%. There was no significant association found between survey year and most frequently requested type of assistance (p = 0.23). However, similar trends were seen to that of importance, where planning and training both increased in the frequency with which they were requested (from 25 to 32% and 21 to 24%, respectively) and consultation and technical support and exercises both decreased in frequency (from 35 to 29% and 14 to 8%, respectively).

## Discussion

Following a system-wide review of preparedness in North Carolina, the state’s regional teams were reorganized by NCDPH to clarify and refine their focus: assisting LHDs with planning, exercises, and training. Other services previously provided by the teams, most notably under the functions of epidemiology and surveillance and public health event response, are now provided through other state offices, including NCDPH’s CDB and OEEB.

In 2009, a core set of 26 services were received by 75% or more of LHDs, whereas in 2012, only 14 services were received by 50% or more of the LHDs. Because of the redistribution of services, it is not surprising that the overall number of services provided by regional offices has declined. However, the results indicate that several services that are still the domain of the regional offices were reported as received by fewer LHDs in 2012 than 2009.

Further exploration is needed to fully understand the reason that significantly fewer LHDs reported receiving these 22 services which fall under the categories of planning, communication and liaison assistance, consultation and technical assistance, and exercise assistance. Some respondents suggested that the decrease in services was due to the larger geographic area of regions and the higher number of counties. Analysis of the data seem to bear this perception out; for 17 services, the proportion of LHDs in a region reporting the service as received decreased significantly as region size increased.

Other factors may have also contributed to the lower number of LHDs reporting that certain services were received. These include the shorter time frame investigated in the 2012 study (6 months) compared to the 2009 study (12 months) and the staffing level and experience of the newly formed teams. Not all of the regional offices were fully staffed and staff that was in place was new to their position as compared to PHRSTs that has been in place for more than 8 years. In addition, NCDPH had requested that regional offices focus on assessing an LHD baseline for the CDC’s Public Health Preparedness Capabilities [9]. The decrease in exercise support, specifically, is most likely attributable to the lack of a requirement for teams to coordinate a regional exercise in 2012, which had been a mandate in 2009.

This study has several limitations. Respondents were only asked to indicate whether or not services were received by either checking “yes” or “no.” Respondents were not asked whether each service was needed or requested. The fact that a LHD reported that assistance was not received cannot definitively be interpreted as an indication that assistance was needed and/or requested but not received. Additionally, no data on the quality of each individual service were collected.

Because the survey focused on a single six month period, the services provided may have been dictated by current needs as well as contract agreement addenda between the state and LHDs; a different year of focus might have identified a somewhat different set of services. As mentioned previously, the shorter time period of focus, and the potential for different contractual agreements, could explain some of the differences between the results of the 2012 and 2009 surveys. For example, NCDPH issued a new state-wide isolation and quarantine plan for LHDs in June 2013, which would not have been in place during the time period addressed by the survey. Finally, the 2013 survey was not modified to account for the redistribution of services to other parts of NCDPH (e.g., CDB and OEEB), which may mean that the survey in part measured the regional offices on services they are no longer assigned to provide.

Lastly, respondents were asked to complete the survey as a representative of their entire LHD; although PCs are generally the key point of contact for LHDs with their regional office, it is possible they were not aware of assistance provided by regional offices to other employees in their health department. In particular, PCs may not have been able to report support and services received by communicable disease and other LHD staff.

## Conclusion

This study provides a unique opportunity to understand the services being provided to LHDs in North Carolina by four newly restructured Public Health Preparedness and Response (PHP&R) regional offices, established as part of a strategic planning process conducted by the North Carolina Division of Public Health (NCDPH) to realign preparedness priorities and essential services with appropriate infrastructure. Since most states developed regional public health preparedness infrastructure after 2001, these findings should be informative to other states as they monitor and evaluate their regional infrastructure.

## Abbreviations

AAR: After action FCReport; CAP: Corrective action plan; CDB: Communicable disease branch; CDC: Centers for disease control and prevention; EBCI: Eastern band of Cherokee Indians; Epi-X: Epidemic information exchange; GIS: Geographic information system; IP: Improvement plan; LHD: Local Health Department; NC DETECT: North Carolina Disease Event Tracking and Epidemiologic Collection Tool; NCDPH: North Carolina Division of Public Health; NC HAN: North Carolina Health Alert Network; NCPERRC: North Carolina Preparedness and Emergency Response Research Center National; OEEB: Occupational and environmental epidemiology branch; PC: Preparedness coordinator; PHEP: Public health emergency preparedness; PHP&R Regional office: Public health preparedness and response regional office (4 regional offices formed in 2011); PHRST: Public Health Regional Surveillance Teams (7 regional teams created post September 11, 2001); SNS: Strategic national stockpile.

## Competing interests

The authors declare that they have no competing interests.

## Authors’ contributions

MM and JAH conceptualized the project and obtained funding. CVD implemented the survey and conducted the data analysis. All authors contributed to writing the manuscript and read and approved the final manuscript.

## Authors’ information

CVD is a doctoral student in the Department of Epidemiology at the University of North Carolina Gillings School of Global Public Health.

MM is a Research Associate at the North Carolina Institute for Public Health at the University of North Carolina Gillings School of Global Public Health.

JAH is a Research Assistant Professor in the Department of Epidemiology and Manager of the Research and Evaluation Unit of the North Carolina Institute for Public Health at the University of North Carolina Gillings School of Global Public Health.
